# The predictability of orthodontic tooth movements through clear aligner among first-premolar extraction patients: a multivariate analysis

**DOI:** 10.1186/s40510-022-00447-y

**Published:** 2022-12-30

**Authors:** Linghuan Ren, Lu Liu, Zhouqiang Wu, Di Shan, Lingling Pu, Yanzi Gao, Ziwei Tang, Xiaolong Li, Fan Jian, Yan Wang, Hu Long, Wenli Lai

**Affiliations:** 1 State Key Laboratory of Oral Diseases, National Clinical Research Center for Oral Diseases, Department of Orthodontics, West China Hospital of Stomatology, Sichuan University, Chengdu, 610041 China; 2 Hospital of Stomatology, Lanzhou University, Lanzhou, Gansu Province China

## Abstract

**Background:**

The purpose was to determine the predictability of tooth movements through clear aligner among premolar extraction patients and to explore the effects of various factors on tooth movements.

**Methods:**

A total of 31 extraction patients (10 males and 20 females; age 14–44) receiving clear aligner treatment (Invisalign) were enrolled in this study. The actual post-treatment models and pre-treatment models were superimposed using the palatal area as a reference and registered with virtual post-treatment models. A paired t test was used to compare the differences between actual and designed tooth movements of maxillary first molars, canines, and central incisors. A multivariate linear mixed model was performed to examine the influence of variables on actual tooth movements.

**Results:**

Compared to the designed tooth movements, the following undesirable tooth movements occurred: mesial movement (2.2 mm), mesial tipping (5.4°), and intrusion (0.45 mm) of first molars; distal tipping (11.0°), lingual tipping (4.4°), and distal rotation of canines (4.9°); lingual tipping (10.6°) and extrusion (1.5 mm) of incisors. Age, crowding, mini-implant, overbite, and attachments have differential effects on actual tooth movements. Moreover, vertical rectangular attachments on canines are beneficial in achieving more predictable canine and incisor tooth movements over optimized attachments. Lingual tipping and extrusion of incisors were significantly influenced by the interaction effects between incisor power ridge and different canine attachments (*p* < 0.05).

**Conclusions:**

Incisors, canines, and first molars are subject to unwanted tooth movements with clear aligners among premolar extraction patients. Age, crowding, mini-implant, overbite, and attachments influence actual tooth movements. Moreover, vertical rectangular attachments on canines are beneficial in achieving more predictable incisor tooth movements over optimized canine attachments.

## Introduction

The last two decades have witnessed a dramatic evolution of clear aligner techniques [1]. Clear aligners appeal to both practitioners and patients for their comfort, aesthetics, and ease of use [2, 3]. With the creativity and innovations built into clear aligners, clear aligners are versatile in managing a wide range of malocclusions, such as deep bite, crossbite, open bite, severe crowding, and skeletal anomalies [4–6]. However, there have been concerns that orthodontic tooth movements are not fully achieved through the use of clear aligners, with varying degrees of predictability of tooth movement [7].

In particular, premolar extractions with subsequent incisor retraction, frequently encountered in orthodontic practice, require meticulous biomechanics design that entails adequate intrusion and palatal root-torquing of incisors [8, 9]. These types of tooth movements are considered to be difficult by a recently published complexity evaluation system for clear aligner—clear aligner treatment complexity assessment tool (CAT-CAT) [10]. The predictability of tooth movements for premolar extraction patients is undesirable. Differences exist between predicted and actual tooth movements [11, 12]. As a recent clinical study revealed, unwanted lingual tipping and extrusion of incisors and mesial tipping of first molars occurred among first-premolar extraction patients with clear aligner, leading to anchorage loss and inadequate incisor retraction [11]. Therefore, overtreatment is proposed to achieve extraction space closure successfully. However, the amount of overtreatment has not reached a consensus, justifying more clinical studies investigating the differences between predicted and actual tooth movements among premolar extraction patients [12]. Furthermore, canines are very important anchorage teeth for incisor tooth movements  [13]. Power ridge, an innovative aligner design, has been claimed to offer effective torquing force on incisors [1]. Nevertheless, the tooth movements of canines and the effects of power ridge on incisor torquing among premolar extraction patients utilizing clear aligners have been poorly understood. Consequently, the biomechanics of extraction space closure still remains unclear.

Therefore, we conducted a clinical study investigating the differences between predicted and actual tooth movements among premolar extraction patients. Furthermore, we explored the effects of various factors (e.g., attachments) on these tooth movements, especially the influence of canines on the predictability of incisor tooth movements.

## Materials and methods

### Participants

The study was approved by the institutional review board and ethics committee at the West China Hospital of Stomatology, Sichuan University (No. WCHSIRB-D-2019-087). Orthodontic patients receiving clear aligner therapy at the Department of Orthodontics, West China Hospital of Stomatology, Sichuan University, were enrolled in this retrospective study from September 2018 to September 2020. The inclusion criteria were as follow: (1) extractions of two maxillary first premolars; (2) no missing permanent teeth; (3) clear aligner therapy; and (4) complete pre- and post-treatment clinical data. The exclusion criteria were as follow: (1) non-compliance; (2) receiving auxiliary orthodontic appliances to facilitate tooth movement, e.g., segmental archwire; (3) severe periodontal diseases; and (4) generalized caries.

### Orthodontic treatment through clear aligner therapy

Clear aligner therapy (Invisalign®, California, USA) was prescribed to resolve anterior crowding and retract anterior teeth. All the participants were instructed to wear clear aligners for at least 22 h/day and to change a new pair of clear aligners every 10 days. For all the patients, the treatment staging was briefly designed as follows. Firstly, following the extraction of premolars, molar anchorage preparation (distal tipping) and canine distalization were designed. Then, canine distalization was continued with simultaneous relief of anterior crowding. Lastly, en masse anterior retraction was designed with or without Class II elastic traction. Specifically, the attachment design is shown in Table 1, including both G6 attachment system and rectangular attachments on canines and molars [14, 15]. Demographic and clinical data were collected regarding age, gender, attachment, crowding, overjet, overbite, and orthodontic mini-implants. Orthodontic mini-implants were inserted between the maxillary second premolars and first molars. Patients were instructed to wear orthodontic elastics from the precision cuts on the aligners corresponding to canines to orthodontic mini-implants or to the buttons on the buccal surfaces of mandibular first molars for the augmentation of maxillary molar anchorage [16].

**Table 1:** Baseline patient characteristics

Characteristics	Mean + SD/*N* (%)
Age (year)	25.4 ± 7.8
Child	7 (22.6%)
Female	21 (67.7%)
Overbite (mm)	2.3 ± 1.8
Overjet (mm)	4.3 ± 2.7
Crowding (mm)	5.4 ± 4.8
Mini-implant	8(25.8%)
Canine attachment	
Vertical attachment	15 (24.2%)
Optimized attachment	47(75.8%)
U6 attachment	
G6	25(43.9%)
Horizontal attachment	13 (22.8%)
Vertical attachment	19 (33.3%)
U1 attachment	
Power ridge	23 (37.1%)

*SD* standard deviation, *U1* upper central incisors, *U6* upper first molars

### Model superimposition

**Fig. 1:**
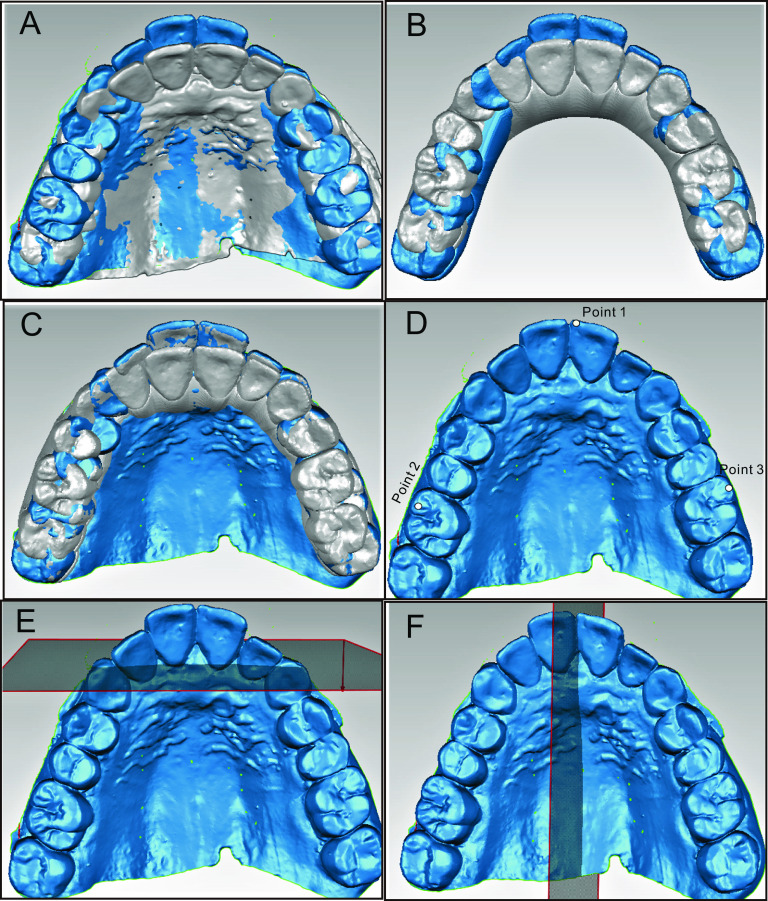
Superimposition of pre- and post-models and establishment of coordinate planes. **A** Best-fit superimposition of actual pre- and post-treatment models based on palatal regions. **B** Virtual pre- and post-treatment models output from ClinCheck. **C** Registration of actual pre-treatment and virtual models. **D** Establishment of the transverse plane. **E** Establishment of the coronal plane; **F** Establishment of the midsagittal plane

Pre- and post-treatment dentition data were scanned and acquired through iTero intraoral scanning (Align Technology, San José, CA, USA). Then, the actual pre- and post-treatment models were imported into Geomagic Studio Software 2014 (Raindrop Geomagio Inc., USA) and superimposed based on the palatal area extending from the third palatal rugae to the palatal vault region (Fig. 1A). Moreover, the virtual pre- and post-treatment models were obtained through ClinCheck software and imported into Geomagic Studio Software (Fig. 1B). The coordinate systems of the actual and virtual models were superimposed by superimposing the actual and virtual pre-treatment models so that the actual and virtual post-treatment models could be compared (Fig. 1C) [11]. The reference planes were established based on the pre-treatment models. Specifically, the transverse plane was defined as a best-fit plane among mesial cusps of bilateral maxillary first molars and the mesioincisal point of the right central incisor (Fig. 1D). The coronal plane went through the midpoint of the incisive papilla and was perpendicular to the transverse plane (Fig. 1E). Furthermore, the midsagittal plane was perpendicular to the transverse and coronal planes and passed through the palatal suture (Fig. 1F).

The linear and angular measurements were performed according to those studies by Luis et al. and Lucchese et al. [17, 18].

### Linear measurements

**Fig. 2:**
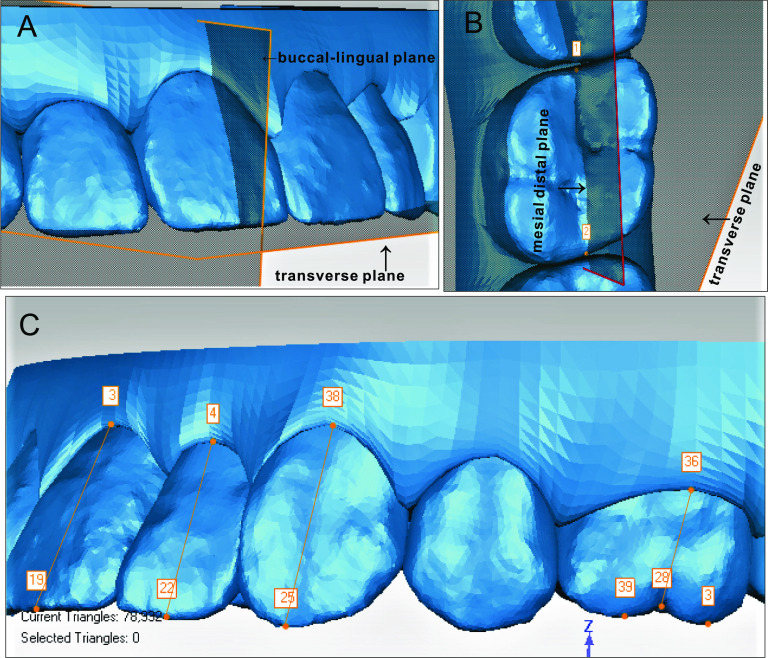
Point digitization and establishment of local tooth plane to measure angles. **A** Establishment of buccal lingual plane of the central incisor. **B** Establishment of mesial–distal plane of the first molar. **C** Point digitization of central incisors, canines and first molars, and axis of central incisors, canines, and first molars

The canines' cusps, the incisor edge's midpoint, and mesial and distal buccal cusps of the upper first molars were digitized as the landmarks to measure tooth movement by calculating the distance between post- and pre-treatment along the sagittal and vertical dimensions through new reference planes, with mesial and extrusive movements defined as positive (Fig. 2C).

### Angular measurements

The mesiodistal angulation and the buccolingual angulation of the tooth axis relative to the reference plane were included in angular measurements, as well as the rotation angle of the canines. For each tooth, an individual coordinate system was established: The individual’s transverse plane was parallel to the global transverse plane; the mesiodistal plane was defined as the plane that was perpendicular to the transverse plane and passed through the mesial and distal points along the central occlusal groove; and the buccolingual plane was the plane that was perpendicular to both the transverse plane and the mesiodistal plane (Fig. 2A, B) [11, 18].

For buccolingual angulation measurement, the tooth axis was projected onto the buccolingual plane to measure buccolingual inclination. The angle between this projected tooth axis and the perpendicular line to the transverse plane was defined as the buccolingual inclination, similar to the mesiodistal angulation measurements. For rotation measurement, the mesial and distal points of the canine occlusal surface were projected onto the transverse plane. The angle between this projected line and the midsagittal plane was defined as the absolute rotation angle. Finally, the difference between actual pre- and post-treatment models was specified as actual tooth movement, while in virtual models was designed as predicted tooth movement [18, 19].

### Statistical analysis

Data on tooth movements from a previous study on Invisalign predictability with a difference between matched pairs of 0.5 mm and a standard deviation of 1.17 mm were used to calculate the sample size [11]. This indicated that 57 samples would be needed to be able to reject the null hypothesis with a power of 80% and a type I error of 0.05. Assuming a small attrition rate, 62 sides requiring bilateral tooth measurement were planned, as each side of the samples had no significant difference. Therefore, 31 patients should be participated [20, 21].

Twenty percent of the patients were randomly selected for re-measurement after two weeks. Intra-class correlation coefficient (ICC) was performed to test the intra-rater reliability. The Kolmogorov–Smirnov test was used to verify the normal distribution of continuous variables. The student’s paired t test was used to compare the differences between the actual and designed tooth movements. A multivariate linear mixed model was performed to examine the influence of variables (age, gender, overbite, overjet, mini-implant, crowding, and attachment treated as a fixed effect) on actual tooth movements, and the predicted tooth movement was defined as a random effect. All the statistical analyses were conducted in SPSS 25.0 (SPSS, Chicago, Illinois, USA) and graphic illustrations were made through Adobe illustrator 2020(Adobe Inc., Mountain View, CA, USA).

## Results

A total of 31 eligible patients were included in this study. The demographic and baseline data of the patients are displayed in Table 1. Briefly, 21 adults and 10 adolescents participated in this study, averaging 25.4 years old. Both optimized and conventional attachments were used on canines and first molars. Orthodontic mini-implants were applied among 8 participants (25.8%) when molar mesial tipping appeared, and power ridge was prescribed in 23 out of 62 central incisors (37.1%).

As shown in Table 2, the intra-class coefficient test revealed that the intra-rater reliability was high (ICC > 0.8), indicative of the reliability of the measurements.

**Table 2:** Intra-class correlation coefficient of tooth measurement

Measurement	ICC	95%CI
*First molar*		
Mesial movement		
Mesial buccal cusp	0.96	0.87, 0.99
Distal buccal cusp	0.85	0.56, 0.95
Extrusion		
Mesial buccal cusp	0.94	0.80, 0.98
Distal buccal cusp	0.86	0.57, 0.96
Mesial tipping	0.90	0.68, 0.97
*Canine*		
Distalization	0.98	0.92, 0.99
Distal tipping	0.87	0.62, 0.96
Lingual tipping	0.89	0.66, 0.97
Distal rotation	0.93	0.78, 0.98
Extrusion	0.91	0.71, 0.97
*Central incisor*		
Retraction	0.95	0.85, 0.99
Extrusion	0.97	0.90, 0.99
Lingual tipping	0.98	0.94, 0.99

*ICC* interclass correlation coefficient

As displayed in Table 3, the first molars' designed tooth movements were observed to have distal tipping (2.9 degrees) with no significant mesial movement or extrusion. However, in reference to the designed tooth movements, the actual tooth movements of the first molars were observed to have mesial movements (2.2 mm for both the mesial and distal cusps, both *p* < 0.001), more intrusion of the mesial cusp (0.45 mm, *p* < 0.01) and more mesial tipping (5.4 degrees, *p* < 0.001) with no significant intrusion of the distal cusp (*p* = 0.84 > 0.05). For canines, the designed tooth movements of canines presented with distalization (6.5 mm) without significant distal tipping, lingual torque, rotation, or extrusion. However, a significant decrease in distal movement of the canines by 1.3 mm was achieved, with a significant increase in distal tipping (10.1 degrees), lingual inclination (5.8 degrees), and distal rotation (5.1 degrees) (all *p* < 0.001) than predicted canine movements. Notably, the extrusion of canines did not differ between the actual and designed tooth movements (*p* = 0.70 > 0.05). The central incisors' actual retraction was 2 mm less than the designed retraction. In contrast, the central incisors achieved more extrusion (1.5 mm) and more lingual inclination (10.6°) than predicted movements (all *p* < 0.001).

**Table 3:** Comparison of actual and designed tooth movements

Measurement^#^	Actual	Designed	Difference	*p* value
*First molar*				
Mesial movement				
Mesial buccal cusp*	2.4 ± 1.8	0.2 ± 1.2	2.2 ± 1.9	< 0.001
Distal buccal cusp*	2.4 ± 1.6	0.2 ± 1.1	2.2 ± 1.7	< 0.001
Extrusion				
Mesial buccal cusp*	− 0.4 ± 0.8	0.1 ± 0.4	− 0.5 ± 0.9	0.001
Distal buccal cusp	− 0.2 ± 0.8	− 0.2 ± 0.6	0.03 ± 1.0	0.837
Mesial tipping*	2.6 ± 7.6	− 2.9 ± 5.7	5.4 ± 7.0	< 0.001
*Canine*				
Distalization*	5.2 ± 2.2	6.5 ± 1.6	− 1.3 ± 1.7	< 0.001
Distal tipping*	10.2 ± 10.8	− 0.9 ± 9.6	11.0 ± 10.7	< 0.001
Lingual tipping*	5.8 ± 6.9	1.4 ± 7.6	4.4 ± 6.6	< 0.001
Distal rotation*	5.1 ± 12.8	0.13 ± 12.74	4.9 ± 9.7	< 0.001
Extrusion*	0.9 ± 1.6	0.9 ± 1.6	0.1 ± 1.2	0.696
*Central incisor*				
Retraction*	3.8 ± 2.0	5.7 ± 2.1	− 2.0 ± 1.8	< 0.001
Extrusion*	1.6 ± 1.3	0.07 ± 1.28	1.5 ± 1.4	< 0.001
Lingual tipping*	10.5 ± 6.6	− 0.2 ± 6.4	10.6 ± 6.6	< 0.001

^#^Data (mean ± SD) are displayed in millimeter (linear data) or degree (angular data)

*p < 0.05 indicates statistical significance

As displayed in Table 4, the multivariate analysis revealed that the extrusion of first molars was significantly associated with age (*β* = 0.96, 95% CI 0.35–1.57; *p* = 0.003), while not with other variables. The mesial tipping of first molars significantly correlated with overbite (*β* = 1.5, 95% CI 0.21–2.79; *p* = 0.023) and was not associated with other predictors. Moreover, the mesial movement of the first molars was significantly associated with crowding (*β* = − 0.25, 95% CI − 0.35 to − 0.15; *p* < 0.0001) and not associated with other factors.

**Table 4:** Linear regression analysis of multiple predictors for actual tooth movement of upper first molars

Predictor	Extrusion		Mesial tipping		Mesial Movement	
	*β* (95%CI)	*p* value	*β* (95%CI)	*p* value	*β* (95%CI)	*p* value
Age						
Adolescent	**0.96 (0.35, 1.57)**	**0.003**	− 0.29 (− 5.69, 5.11)	0.91	− 0.53 (− 1.74, 0.67)	0.38
Adult	Referent		Referent		Referent	
Gender						
Female	− 0.19 (− 0.69, 0.3)	0.44	− 0.41 (− 4.8, 3.98)	0.85	0.88 (− 0.10, 1.86)	0.08
Male	Referent		Referent		Referent	
Crowding	0.039 (− 0.01, 0.09)	0.12	− 0.35 (− 0.76, − 0.06)	0.09	− **0.25 (**− **0.35, **− **0.15)**	** < 0.001**
Mini-implant	0.06 (− 0.54, 0.65)	0.85	3.02 (− 2.3, 8.34)	0.26	− 0.01 (− 1.19, 1.17)	0.99
Overbite	− 0.09 (− 0.23, 0.06)	0.22	**1.5 (0.21, 2.79)**	**0.023**	− 0.09 (− 0.23, 0.06)	0.22
Overjet	0.002 (− 0.12, 0.12)	0.97	0.06 (− 1.05, 1.16)	0.91	0.002 (− 0.12, 0.12)	0.97
Attachment						
Horizontal	0.16 (− 0.38, 0.71)	0.55	− 0.71 (− 5.59, 4.17)	0.77	− 0.06 (− 1.15, 1.02)	0.91
G6	0.17 (− 0.35, 0.68)	0.52	− 4.42 (− 8.96, 0.12)	0.06	− 0.05 (− 1.06, 0.96)	0.92
Vertical	Referent		Referent		Referent	

Bold values indicate statistical significance (*p* < 0.05)

Designed: designed tooth movement

As presented in Table 5, the extrusion of canines was significantly associated with crowding (*β* = 0.14, 95% CI 0.08–0.21; *p* < 0.001) while not related to other predictors.

**Table 5:** Linear regression analysis of multiple predictors for actual tooth movement of canines

Predictor	Extrusion		Distal tipping		Distal movement	
	*β* (95%CI)	*p* value	*β* (95%CI)	*p* value	*β* (95%CI)	*p* value
Age						
Adolescent	0.70 (− 0.03, 1.44)	0.06	− 1.43 (− 7.34, 4.49)	0.63	0.12 (− 1.04, 1.29)	0.83
Adult	Referent		Referent		Referent	
Gender						
Female	0.61 (− 0.04, 1.25)	0.06	1.95 (− 3.14, 7.04)	0.45	0.41 (− 0.63, 1.46)	0.43
Male	Referent		Referent		Referent	
Crowding	**0.14 (0.08, 0.21)**	** < 0.001**	0.15 (− 0.30, 0.60)	0.51	**0.13 (0.04, 0.23)**	**0.008**
Mini-implant	− 0.42 (− 1.08, 0.24)	0.21	**11.53 (6.57, 16.49)**	** < 0.001**	0.62 (− 0.42, 1.66)	0.24
Overbite	0.07 (− 0.09, 0.23)	0.36	− 0.72 (− 1.95, 0.51)	0.24	− 0.13 (− 0.40, 0.14)	0.33
Overjet	0.01 (− 0.11, 0.13)	0.84	**1.09 (0.20, 1.99)**	**0.02**	**0.22 (0.03, 0.41)**	**0.02**
Attachment						
Optimal	0.18 (− 0.44, 0.80)	0.56	**4.92 (0.13, 9.70)**	**0.04**	0.93 (− 0.07, 1.93)	0.07
Vertical	Referent	Referent	Referent	Referent	Referent	Referent

Bold values indicate statistical significance (*p* < 0.05)

Designed: designed tooth movement

The distal tipping of canines was significantly correlated with mini-implant (*β* = 11.53, 95% CI 6.57–16.49; *p* < 0.001), overjet (*β* = 1.09, 95% CI 0.20–1.99; *p* = 0.02 < 0.05), and canine-optimized attachments (*β* = 4.92, 95% CI − 0.13–9.70; *p* = 0.04 < 0.05). However, it was not correlated with other variables. Moreover, the results revealed that the distal movement of canines was significantly associated with crowding (*β* = 0.13, 95% CI 0.04–0.23; *p* = 0.008) and overjet (*β* = 0.22, 95% CI 0.03–0.41; *p* = 0.02 < 0.05), and not with other predictors.

As displayed in Table 6, we found that the extrusion of central incisors was significantly associated with overjet (*β* = 0.16, 95% CI 0.02–0.30; *p* = 0.03 < 0.05) and canine attachments (*β* = 0.94, 95% CI 0.2–1.68; *p* = 0.01 < 0.05). The lingual tipping of central incisors was significantly correlated with crowding (*β* = − 0.47, 95% CI − 0.79 to − 0.14; *p* = 0.006). However, it was not associated with other predictors. Moreover, the retraction of central incisors was not associated with other predictors.

**Table 6:** Linear regression analysis of multiple predictors for actual tooth movement for incisors

Variables	Extrusion		Lingual tipping		Retraction	
	*β* (95%CI)	*p* value	*β* (95%CI)	*p* value	*β* (95%CI)	*p* value
Age						
Adolescent	0.24 (− 0.63, 1.12)	0.58	− 3.62 (− 7.49, 0.26)	0.07	− 0.96 (− 2.12, 0.20)	0.10
Adult	Referent		Referent		Referent	
Gender						
Female	0.22 (− 0.55, 0.99)	0.57	− 0.45 (− 4.08, 3.19)	0.81	− 0.36 (− 1.39, 0.67)	0.49
Male	Referent		Referent		Referent	
Crowding	0.04 (− 0.04, 0.11)	0.32	− **0.47 (**− **0.79, **− **0.14)**	**0.006**	− 0.02 (− 0.13, 0.08)	0.65
Mini-implant	0.25 (− 0.52 1.02)	0.52	1.49 (− 2.17, 5.15)	0.42	− 0.08 (− 1.13, 0.98)	0.89
Over bite	− 0.16 (− 0.42, 0.11)	0.24	− 0.57 (− 1.52, 0.38)	0.23	− 0.10 (− 0.36, 0.16)	0.45
Over jet	**0.16 (0.02, 0.30)**	**0.03**	0.43 (− 0.22, 1.09)	0.20	0.10 (− 0.09, 0.29)	0.31
Attachment	–	–	–	–		
Power Ridge	− 0.45 (− 1.07, 0.18)	0.16	− 0.008 (− 3.37, 3.35)	0.19	− 0.26 (− 1.11, 0.59)	0.54
No*	Referent		Referent		Referent	–
Canine attachment						
Optimal	**0.94 (0.20, 1.68)**	**0.01**	3.28 (− 0.14, 6.70)	0.06	0.78 (− 0.20, 1.77)	0.12
Vertical	Referent		Referent		Referent	

Bold values indicate statistical significance (*p* < 0.05)

Result from the multiple linear regression analysis for actual tooth movement of central incisors

*p* value and 95% confidence interval (CI) ranges of the coefficient for each variable. The coefficient (*β*) presents the effect strength of the respective factor. *p* value below 0.05 was considered as statistical significance

**Fig. 3:**
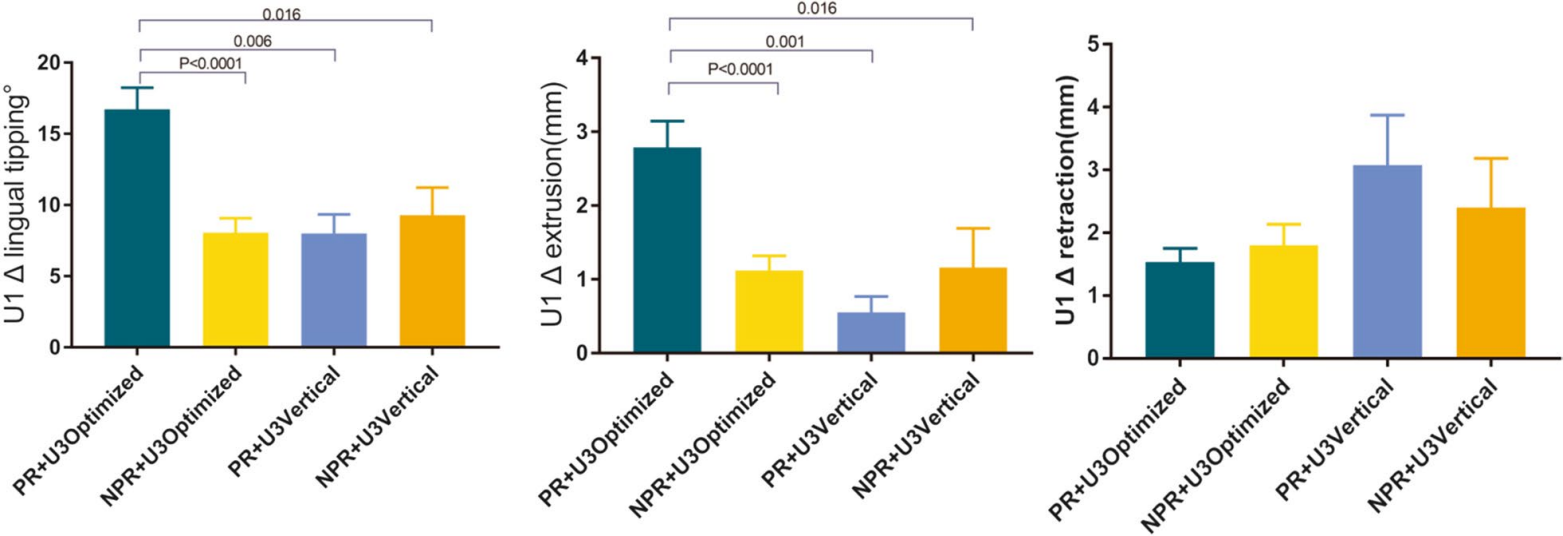
Variation of upper incisor movements using different canine and incisor attachments. PR, power ridge used on central incisors; NPR, no power ridge on central incisors; U3 Optimized, optimized attachments used on canines; U3 Vertical, vertical attachments used on canines. Δlingual tipping, Δextrusion and Δretraction indicate difference between actual and designed tooth movement. Tukey’s post hoc test was used for pairwise comparison between groups of different attachments with the same variance

Furthermore, as displayed in Fig. 3, the differences between the actual and designed tooth movements of central incisors (Δ U1 lingual tipping and Δ U1 extrusion) were significantly influenced by the interaction effects between power ridge on incisors and canine attachments. Specifically, central incisors were more lingually tipped and extruded with power ridge + canine-optimized attachments as compared to the other three subgroups (power ridge + canine vertical rectangular attachment; no power ridge + canine-optimized attachment; no power ridge + canine vertical rectangular attachment) (all *p* < 0.05). However, the retraction of incisors was not influenced by the interaction effects of power ridge and canine attachments (*p* > 0.05).

## Discussion

Although clear aligner offers advantages of comfort, esthetics, and ease of oral hygiene care over its counterpart—fixed appliance [2, 3], its treatment efficacy has been of concern due to varying predictability of tooth movement [7], especially anterior retraction following premolar extractions [16]. From the perspectives of aligner biomechanics, mesial tipping of molars, distal tipping of canines, and lingual tipping and extrusion of incisors may occur since the retraction force (on anterior teeth) and protraction force (on molars) pass occlusally to their corresponding centers of resistance. Refinement or segmental archwire aiming to distalize molars and intrude incisors may be prescribed for this clinical situation [8]. Some clinical studies have demonstrated this phenomenon [11, 12]. As shown in our study, first molars were more mesialized by 2.2 mm with more mesial tipping of 5.4 degrees compared to the designed tooth movements. However, overtreatment (distal tipping) of first molars (2.9 degrees) was prescribed to counter the anchorage loss of first molars. Meanwhile, the distal cusp of the first molars remained stable in the vertical dimension, but the mesial cusp of the first molars was more intruded by 0.45 mm. This could explain the clinical phenomenon of buccal open bite in premolar extraction cases during clear aligner treatments [11, 12]. Thus, to avoid the anchorage loss of molars, more molar distalization may be prescribed to offset the mesial movement of molars.

Our results revealed that, compared to the designed tooth movements, canines were less distalized with more distal tipping, lingual tipping, mesial rotation, and extrusion. In particular, the lingual tipping of canines renders the canine roots to contact labial alveolar cortex and impedes canine root movement, resulting in distal tipping (if only root apices contact the alveolar cortex) or even no movement (if the whole roots contact the alveolar cortex) [22]. Moreover, severe lingual tipping risks canine bone fenestration. Thus, additional lingual root-torquing of canines should be prescribed to prevent these adverse effects.

As previous studies showed, unwanted lingual tipping and extrusion of incisors were encountered [8, 11]. This could lead to incisor interference and, together with mesial tipping and intrusion of first molars, result in anterior deep bite and posterior open bite, a phenomenon frequently encountered in clinical practice [23]. As mentioned above, as the overbite deepens, the incisor interference prevents the anterior teeth from being retracted, resulting in a greater molar mesial tipping if patients continue to change new pairs of aligners. In addition, we found that the actual mesial tipping of first molars was significantly associated with an overbite (*β* = 1.5). This indicates that additional 1.5 degrees of mesiodistal tipping of first molars will occur if overbite is increased by one millimeter. Furthermore, it suggests that deep overbite may lead to mesial inclination of molars owing to interference of anterior teeth and eventually results in failure of space closure. Thus, unwanted lingual tipping (torque loss) and extrusion of incisors should be avoided for extraction cases, and leveling the curve of Spee is of paramount importance to premolar extraction cases with clear aligners. Importantly, there are several variables that can affect the predictability of tooth movement, which we discuss below.

### Crowding

As demonstrated in recent research, less space remains after the resolution of anterior crowding among patients with more anterior crowding [24]. Thus, the amount of en masse incisor retraction may be reduced for patients with more anterior crowding, thereby reducing the likelihood of molar anchorage loss since en masse incisor retraction requires greater molar anchorage [24]. Consistently, the multivariate analysis revealed that the actual mesial movement of first molars was significantly associated with crowding (*β* = − 0.25), which means that an increase in crowding by 1 mm would decrease the mesial movement of first molars by 0.25 mm. We found that crowding was negatively associated with lingual tipping of central incisors (*β* = − 0.47). This finding could be partly explained by the fact that more crowding is associated with less bodily retraction of incisors, resulting in less lingual tipping of the incisors. Meanwhile, we found that the actual distal movement of canines was positively associate with crowding (*β* = 0.13) and extrusion (*β* = 0.14) of canines. Therefore, this could be attributed to the fact that greater canine distalization was required for space gaining to solve anterior crowding, resulting greater canine extrusion.

### Age and overjet

We also found that the actual extrusion of first molars was significantly associated with age (*β* = 0.96). These results indicate that a change from an adult patient to an adolescent patient will increase the extrusion of first molars by 0.96 mm, possibly due to the eruption of first molars among adolescents. Overjet was positively associated with distal movement and the distal tipping of canines, which could be explained by greater amounts of distal movement and subsequent distal tipping of canines required for correcting a larger overjet. Furthermore, a larger overjet requires a greater amount of incisor retraction and causes a greater amount of incisor extrusion, which likely explains the result that the extrusion of central incisors was positively associated with overjet (*β* = 0.16).

### Mini-implant

The clinical effectiveness of orthodontic mini-implants has been well documented for preserving molar anchorage fixed appliances [25, 26]. Ironically, our results revealed that using mini-implants did not help preserve molar anchorage, likely due to the mode of mini-implant application and the influence of anterior interference. For clear aligner treatment, the molar anchorage is often augmented by wearing elastics between the precision cuts on canines and the mini-implants, with no direct force acting on posterior teeth for anchorage augmentation. Apart from that, mini-implants were placed when the mesial movement of the first molars had occurred. In this situation, molars are still susceptible to mesial movement and tipping even if mini-implants are used, especially when anterior incisor interference is present. Regardless of the magnitude of retraction force offered by the mini-implants, the incisor interference prevents the upper incisors from retracting and results in molar tipping if new pairs of aligners are worn [27]. Importantly, the results showed that mini-implant was positively associated with distal tipping (*β* = 11.53) of canines, which could be due to greater distalization force that was exerted on canines by mini-implants.

### Attachment

It has been claimed that the Invisalign G6 system is beneficial for preserving molar anchorage due to optimized attachments on first molars that offer additional distalization force [1]. Additionally, it has been suggested to be beneficial for root control due to the additional anti-tipping moments provided by the optimized attachments on canines [1, 23, 27]. However, our results revealed that the mesial movement and extrusion of first molars did not differ among different types of attachments on first molars, except that the mesial tipping of first molars decreases with Invisalign G6 attachments (*β* = − 4.42, 95% CI − 8.96–0.12; *p* = 0.06 < 0.1). In contrast, compared to the vertical rectangular attachments, we found that canine-optimized attachments are associated with more distal tipping of canines (*β* = 4.92). Thus, we suggest vertical rectangular attachments on canines are superior to optimized attachments on canine root control among extraction cases.

Furthermore, the power ridge was invented to offer additional lingual root torque on incisors and is claimed to avoid lingual tipping and extrusion of incisors [23, 28]. However, the clinical effectiveness of the power ridge in achieving predicted lingual root torque was investigated in a clinical study by Simon et al. [29], where no difference in incisor torque was found with and without the power ridge. Likewise, we found that the power ridge did not influence the lingual tipping, extrusion, and retraction of central incisors on central incisors, which is in line with the previous study [30].

**Fig. 4:**
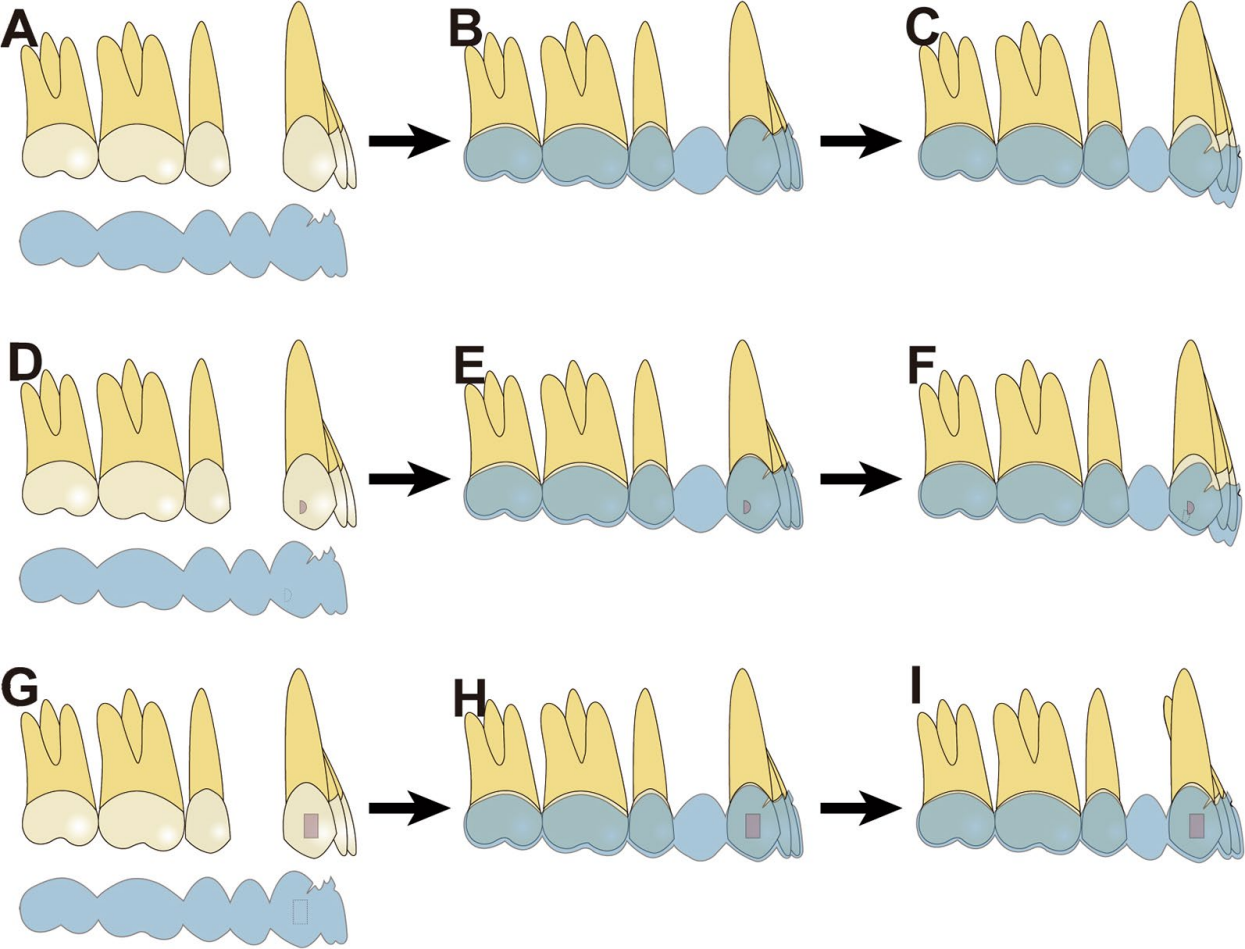
Effects of canine attachments on incisor retraction. **A** No attachment on canines. Before wearing aligner. **B** The aligner was put onto the dentition. **C** Due to the retraction force and the additional lingual root-torquing force, the aligner tended to move incisally. **D**–**F** Optimized attachment on canine. Due to the inadequate retention offered by optimized attachments, the aligner tended to move incisally. **G**–**I** Vertical attachment on canine. Due to the adequate retention offered by the bulky vertical attachment, the aligner was retained in position

Interestingly, we found that unfavorable lingual tipping (*β* = 3.28) and extrusion (*β* = 0.94) of central incisors were associated with optimal attachments on canines (with vertical attachments on canines being the referent). These findings suggest that canines may be the principal teeth that offer anchorage for incisor control when retracting anterior, consistent with the result of Liu et al. [13]. As mentioned above, aligners tend to have incisal movement when designed for lingual root-torquing. Thus, adequate retention of clear aligners on incisors and canines is required to express these desired tooth movements. Due to the more significant bulkiness of vertical rectangular attachments, as compared to optimized attachments, the retention of clear aligners is more favorable with vertical rectangular attachments than with optimized attachments, resulting in better aligner retention on both the anchorage teeth (canines) and the teeth to be moved (incisors) [13]. This leads to a higher expression of lingual root-torquing and intrusion of incisors. Moreover, we were curious about the interaction effect between incisor power ridge and canine attachments. Even more so, as displayed in Figs. 3 and 4, when the power ridge was designed on incisors, undesirable lingual tipping and extrusion are less with canine vertical rectangular attachments compared to canine optimized attachments. Thus, we suggest the power ridge be accompanied by vertical rectangular attachments on canines to reinforce incisor intrusion and lingual root-torquing control.

As previous studies have shown that the medial 2/3 of the third rugae and the regional palatal vault dorsal might be stable regions to register 3D digital models for evaluation of orthodontic tooth movement in adult patients, we chose this region as a relatively stable structure to evaluate tooth movement [31]. Specifically, 4 unloaded miniscrews were used as stable structures to evaluate the reliable region throughout orthodontic treatment [31]. Although a skull region based on CBCT for superimposition models is considered to be more stable, its clinical application is limited by greater exposure to radiation. Meanwhile, a 3D CBCT examinations for model evaluation might be more suitable than a 2D cephalometric measurement. However, the difference in the vertical dimension should be interpreted with caution, especially in adolescents [17].

This study had some notable limitations. Firstly, more prospective studies with larger sample sizes are required for further analysis of valuable variables as this was a retrospective study with limited sample sizes. Secondly, the way mini-implants are used can impact the results. In our present study, mini-implants were placed when the mesial movement of the first molars had occurred, so molars were still susceptible to mesial movement and mesial tipping even if mini-implants were used. Therefore, the effect of mini-implant was possibly underestimated.

## Conclusions

1. Among premolar extraction cases treated with clear aligners, the following undesirable tooth movements tend to occur: mesial movement, mesial tipping, and intrusion of first molars; distal tipping, lingual tipping, and distal rotation of canines; lingual tipping and extrusion of incisors. Thus, appropriate overtreatment may be designed to eliminate these unwanted tooth movements.
2. Age (adolescent vs. adult), crowding, mini-implant, overbite, overjet, and attachments had differential effects on actual tooth movements of extraction space closure.
3. Canines are important anchorage teeth required for achieving torque control and vertical control of incisors.
4. When power ridges are prescribed on incisors, lingual root-torquing and intrusion of incisors are more predictable with vertical rectangular attachments than with optimized attachments on canines.

## Data Availability

Not applicable.
